# Microbiota, Food, and Health

**DOI:** 10.3390/ijms20246329

**Published:** 2019-12-15

**Authors:** Kirsi Laitinen, Miguel Gueimonde

**Affiliations:** 1 Institute of Biomedicine, University of Turku, FI-20014 Turku, Finland; 2 Instituto de Productos Lácteos de Asturias (IPLA), CSIC, 33300 Asturias, Spain

**Keywords:** intestinal microbiota, food, diet, health

The effects of specific foods, such as products containing probiotics or prebiotics, on human health and the role of intestinal microbiota in this interaction have been a subject of scientific interest for several decades. However, it was not until recently that, largely due to the development of next-generation sequencing techniques, we finally began to understand the full complexity of intestinal microbiota. During the last decade, several research projects and publications have demonstrated that microbiota play a key role in maintaining health, and that microbiota dysbiosis is involved in different communicable and non-communicable diseases. It has become clear that diet is a major driver of microbiota composition and function, and the research in this area is thus of utmost importance. However, our knowledge on the impact of specific foods, nutrients, and dietary patterns upon the intestinal microbiota at different life stages is still limited. We do not fully understand the role of the microbiota–food interaction in the maintenance of human health and are just starting to identify the differential effects of both long- and short-term dietary interventions on intestinal microbiota in different age groups. Moreover, the influence of gut microbiota in the transformation of dietary components and the concomitant effects on the bioavailability of nutrients, bioactivity, and health have only been elucidated for a very limited number of dietary compounds. In this context, this “Microbiota, Food, and Health” issue covers different aspects of this microbiota–diet interaction by including research articles and reviews comprising both in vitro and in vivo evidence on the interplay between diet and microbiota. 

This Special Issue considers different aspects of the microbe–food–health interaction, from molecular to intervention studies, including both animal and human data. In their molecular study, Rong and coworkers [1] take advantage of domain swapping and molecular docking for characterizing the structural basis of the substrate specificity of fungal ω-3 fatty acid desaturases, involved in the biosynthesis of long-chain PUFA. Moving from the most basic molecular studies to the area of food processing and dietary xenobiotics, Nogacka and collaborators [2] also review in this Special Issue the evidence on the formation of carcinogens during meat cooking, its impact in colorectal cancer risk, and the potential interaction of these compounds with intestinal microbiota. The potential strategies for diminishing the effect of these xenobiotics, for instance, by using probiotics and/or prebiotics for the modulation of gut microbiota, are also discussed. To this regard, different in vitro systems have been used for assessing the interaction between nutrients and the microbiota. In their manuscript, Tsiko and coworkers [3] use a miniaturized fermentation model for assessing the impact of different dietary fibers upon human fecal microbiota. The authors report on the changes induced by different rye and oat fiber preparations on the microbiota and microbial metabolites and on the amelioration of the deleterious impact of antibiotics on the intestinal ecosystem. 

The Special Issue also includes studies on the microbiota of production animals. The microbiota of sows was characterized by means of 16S rRNA gene-based profiling and found to be different from that of growers in the study by Niu and colleagues [4]. Moreover, the authors observed a positive association between some intestinal microorganisms and the apparent nutrient digestibility. In another study, also performed in pigs, Adhikari and collaborators [5] used the same technique for assessing the microbiota composition in different locations, including lumen and mucosa, along the intestinal tract of weaned piglets. The results underline the microbiota changes occurring during the month following weaning, as well as the presence of clearly different microbial communities in the different regions of the intestinal tract.

With regard to the human studies reported in this Special Issue, Cortes and coworkers [6] demonstrate, in their observational study, that metaproteomics constitute a feasible approach to investigate the global protein and pathway expression in fecal samples. This approach in combination with 16s rRNA gene profiling allowed the detailed study of the infant microbiome and the evaluation of the functional community-wide metabolic status of the infants representing different gut microbial environments with regard to factors including delivery or feeding mode and use of antibiotics. Jalanka and coworkers [7] addressed, in a double-blind study setting, the impact of dietary psyllium consumption with anticipated prebiotics effects on fecal microbiota composition as well as gut environmental conditions in participants with and without constipation. In this Special Issue article, psyllium was found to increase fecal water and to induce changes in gut microbiota composition. A more global approach was taken in the study by Laitinen and Mokkala who addressed diet–microbiota relations [8]. They demonstrated that a higher index of diet quality, in other words food consumption in accordance with dietary recommendations, was related to higher gut microbiota diversity. This kind of approach of measuring dietary quality with a stand-alone “simple to use and analyze” tool could be a minimum requirement for controlling dietary intake in gut microbiome studies. The approach also encourages health counselling towards healthy eating with simultaneous benefits for gut microbiome. In the review article by Ganesan and collaborators [9], the literature on the relation of diet and gut microbiota as well as that of gut microbiota and diabetes, a metabolic condition with an increasing prevalence, are highlighted. The review focuses particularly on *Faecalibacterium prausnitzii* and its potential use as a diagnostic and therapeutic marker. In the review by Wegh and coworkers [10], the definition of postbiotics and their potential applications in the context of microbe–host interaction are discussed. Postbiotics include non-viable microbial cells that may act through several proposed mechanisms in health modulation, an example being the induction of the host immune system. 

Overall, this Special Issue provides new data and novel insights into the complex interactions that take place in our gut, where the dietary components, the residing microbiota, and host cells get together in a way that becomes critical for the maintenance of health. The manuscripts included in the Special Issue also point out the future challenges that, if addressed, promise to expand our future understanding on the trinomial microbiota–food–health interaction.
